# Fixing a Shaky Video to Remotely Program Deep Brain Stimulation

**DOI:** 10.5334/tohm.963

**Published:** 2025-07-28

**Authors:** Maria Belen Justich, Alexandra Boogers, Andres M. Lozano, Alfonso Fasano

**Affiliations:** 1 Edmond J. Safra Program in Parkinson’s disease and Morton and Gloria Shulman Movement Disorders Centre, Toronto Western Hospital, UHN and Division of Neurology, University of Toronto, Toronto, Ontario, Canada; 2 Department of Neurosurgery, University of Toronto, Toronto, Ontario, Canada; 3 Krembil Brain Institute, Toronto, ON, Canada; 4 CenteR for Advancing Neurotechnological Innovation to Application (CRANIA), Toronto, ON, Canada

**Keywords:** Deep brain stimulation, Parkinson’s disease, Tremor, Remote Programming, Telemedicine

## Abstract

Deep brain stimulation (DBS) is an increasingly utilized therapy for treating refractory tremor in Parkinson’s disease (PD). Remote care can improve patient access to specialized DBS clinics. Here, we present a novel strategy to assess tremor remotely during DBS programming.

We report the case of a 65-year-old female diagnosed with PD who showed only partial responsiveness to levodopa. She underwent bilateral subthalamic nucleus DBS surgery and was implanted with an Infinity™ implantable pulse generator with directional leads (Abbott, Chicago, IL, USA). Given that she resided 1,500 km from our center, device programming was performed remotely using the Neurosphere™ Virtual Clinic platform. The patient was instructed to hold her controller in a fixed position until her resting tremor re-emerged, which was visibly evident through a shaking video frame. Stimulation parameters were then optimized until the video frame became still. She reported sustained benefit during follow-up.

We propose that this alternative method for remotely assessing upper limb tremor may offer advantages for healthcare providers, allowing them to base stimulation adjustments on visually observable tremor severity.

Deep Brain Stimulation (DBS) is an increasingly used therapy to treat refractory tremor in Parkinson’s disease (PD) patients [1]. Recurrent postoperative appointments for programming tuning are fundamental for successful outcomes [2]. In Canada, long distances and difficult weather conditions may restrict the access to DBS-specialized clinics. Novel technologies, particularly remote programming, may overcome these issues [3]. We aimed to describe a new strategy to assess tremor remotely during DBS programming.

We present the case of a 65-year-old female patient with a 7-year-history of tremor-dominant PD. Despite receiving a daily dose equivalent to 1620 mg of levodopa, her tremor improved by <40% during the levodopa challenge, as assessed by the resting tremor items of the Unified Parkinson’s Disease Rating Scale (UPDRS) Part III. A multidisciplinary preoperative evaluation—including Psychiatry, Neuropsychology, Neurology, and Neurosurgery—was conducted. She demonstrated a good response to a supramaximal dose of levodopa, with her overall UPDRS-III score improving from 52 to 28. Given her motor fluctuations and dyskinesias, and in the absence of concerning neuropsychiatric or cognitive symptoms, she was deemed a suitable candidate for bilateral subthalamic nucleus (STN) DBS surgery. The patient underwent successful surgery and was implanted with an Infinity™ implantable pulse generator (IPG) with directional leads, by Abbott (Chicago, IL, USA). As her residence was located 1,500 km from our center, device programming was performed remotely using the Neurosphere™ virtual clinic platform.

Two months after surgery, DBS initial programming session was carried out at our center. After identifying the therapeutic thresholds for each contact in person, the patient settings were fine-tuned remotely on a weekly basis. During the evaluation, the patient was asked to hold her controller in a fixed position until her rest tremor re-emerged, one hand at a time. Once tremor reached the maximum amplitude and was clearly noticeable by a shaking video-frame, we optimized the stimulation parameters by increasing amplitude and frequency, until the frame was still (video 1). The final settings for her left STN were C+11a-11c-, 3.5mA, 60µs, 160Hz. She reported a sustained benefit in the follow-up, and her levodopa equivalent dose was gradually reduced by 60% compared to baseline.

**Video 1:** **DBS programming guided by a shaky videoframe.** Remote programming to address right hand resting tremor. Part 1 shows baseline tremor, Part 2 optimization process and Part 3 the final result.

To date, the use of remote care to assess and treat PD patients remains controversial. Although some authors recommend in-person appointments for initial programming after DBS surgery, growing evidence supports that satisfactory outcomes can be reached with telemedicine [4,5]. Tremor, a core feature of PD, is among the symptoms that can be assessed remotely. However, this can be challenging, as patients may struggle to position themselves appropriately within the camera frame, or image resolution may be insufficient to detect subtle tremors. We propose that this alternative method of assessing upper limb tremor may offer advantages—not only for patients, who are only required to hold the DBS controller, but also for healthcare providers, who could base their adjustments on more visible shakiness. Nevertheless, our report is limited to a single case. A prospective cohort comparing different strategies will be needed to validate our findings.

## Financial disclosures

This work was supported by the Chair in Neuromodulation (AF) at University of Toronto and University Health Network, Toronto, ON, Canada.

MBJ: declares that there are no disclosures to report.

AB: reports receiving honoraria from American Academy of Neurology and Abbvie, consultancy fees from Abbvie, Abbott and Boston Scientific, and research grant from Fernand Lazard Foundation. All disclosures are not relevant to the paper.

AML: is a consultant to Abbott, Boston Scientific, Insightec, Medtronic. AL is a Scientific Director at Functional Neuromodulation.

AF: is a consultant to Abbvie, Ceregate, Medtronic, Boston Scientific, Iota, Inbrain, Inbrain Pharma. AF received honoraria from Abbvie, Medtronic, Boston Scientific, Sunovion, Chiesi farmaceutici, UCB, Ipsen; grants from University of Toronto, Weston foundation, Abbvie, Medtronic, Boston Scientific, CIHR.

## References

[1] Deuschl G, Antonini A, Costa J, Ś;miłowska K, Berg D, Corvol J-C, et al. European Academy of Neurology/Movement Disorder Society-European Section Guideline on the Treatment of Parkinson’s Disease: I. Invasive Therapies. Mov Disord. 2022;37:1360–1374. DOI: 10.1002/mds.29066 35791767

[2] Munhoz RP, Albuainain G. Deep brain stimulation: new programming algorithms and teleprogramming. Expert Rev Neurother. 2023 May;23(5):467–478. Epub 2023 May 3. PMID: 37115193. DOI: 10.1080/14737175.2023.2208749 37115193

[3] Fung WKW, Justich MB, Hamani M, Munhoz RP, Kalia SK, Lozano AM, Fasano A. Remote Deep Brain Stimulation Programming in Canada. Movement Disorders Clinical Practice; 2024. DOI: 10.1002/mdc3.13999 PMC11078478 38369588

[4] Sharma VD, Safarpour D, Mehta SH, Vanegas-Arroyave N, Weiss D, Cooney JW, Mari Z, Fasano A. Telemedicine and Deep brain stimulation – Current practices and recommendations. Parkinsonism Relat Disord. 2021 Aug 1;89:199–205. DOI: 10.1016/j.parkreldis.2021.07.001 34274215

[5] Jitkritsadakul O, Rajalingam R, Toenjes C, Munhoz RP, Fasano A. Tele-health for patients with deep brain stimulation: The experience of the Ontario Telemedicine Network. Mov Disord. 2018 Mar 1;33(3):491–492. DOI: 10.1002/mds.27230 29119600

